# Advancing the literature on designing audit and feedback interventions: identifying theory-informed hypotheses

**DOI:** 10.1186/s13012-017-0646-0

**Published:** 2017-09-29

**Authors:** Heather L. Colquhoun, Kelly Carroll, Kevin W. Eva, Jeremy M. Grimshaw, Noah Ivers, Susan Michie, Anne Sales, Jamie C. Brehaut

**Affiliations:** 1 Department of Occupational Science and Occupational Therapy, University of Toronto, 160-500 University Ave, Toronto, Ontario M5G 1V7 Canada; 2 Ottawa Hospital Research Institute, Clinical Epidemiology Program, The Ottawa Hospital, General Campus, Centre for Practice Changing Research, 501 Smyth Road, Ottawa, Ontario K1H 8L6 Canada; 3 Centre for Health Education Scholarship, Department of Medicine, University of British Columbia, Vancouver, BC V5Z 4E3 Canada; 4 Department of Medicine, University of Ottawa, 451 Smyth Road, Ottawa, Ontario K1H 8M5 Canada; 5 Department of Family Medicine, Women’s College Hospital, Toronto, ON M5S 1B2 Canada; 6 Department of Clinical, Educational and Health Psychology, London, University College London, London, WC1E 6BT UK; 7 Department of Learning Health Sciences, University of Michigan Medical School, VA Ann Arbor Healthcare System, Health Services Research and Development, Ann Arbor, MI 48109 USA; 8 School of Epidemiology, Public Health and Preventive Medicine, University of Ottawa, 451 Smyth Road, Ottawa, Ontario K1H 8M5 Canada

**Keywords:** Audit and feedback, Implementation science, Knowledge translation, Theory

## Abstract

**Background:**

Audit and feedback (A&F) is a common strategy for helping health providers to implement evidence into practice. Despite being extensively studied, health care A&F interventions remain variably effective, with overall effect sizes that have not improved since 2003. Contributing to this stagnation is the fact that most health care A&F interventions have largely been designed without being informed by theoretical understanding from the behavioral and social sciences. To determine if the trend can be improved, the objective of this study was to develop a list of testable, theory-informed hypotheses about how to design more effective A&F interventions.

**Methods:**

Using purposive sampling, semi-structured 60–90-min telephone interviews were conducted with experts in theories related to A&F from a range of fields (e.g., cognitive, health and organizational psychology, medical decision-making, economics). Guided by detailed descriptions of A&F interventions from the health care literature, interviewees described how they would approach the problem of designing improved A&F interventions. Specific, theory-informed hypotheses about the conditions for effective design and delivery of A&F interventions were elicited from the interviews. The resulting hypotheses were assigned by three coders working independently into themes, and categories of themes, in an iterative process.

**Results:**

We conducted 28 interviews and identified 313 theory-informed hypotheses, which were placed into 30 themes. The 30 themes included hypotheses related to the following five categories: A&F recipient (seven themes), content of the A&F (ten themes), process of delivery of the A&F (six themes), behavior that was the focus of the A&F (three themes), and other (four themes).

**Conclusions:**

We have identified a set of testable, theory-informed hypotheses from a broad range of behavioral and social science that suggest conditions for more effective A&F interventions.

This work demonstrates the breadth of perspectives about A&F from non-healthcare-specific disciplines in a way that yields testable hypotheses for healthcare A&F interventions. These results will serve as the foundation for further work seeking to set research priorities among the A&F research community.

**Electronic supplementary material:**

The online version of this article (10.1186/s13012-017-0646-0) contains supplementary material, which is available to authorized users.

## Background

Audit and feedback (A&F), where data about specific aspects of practice are summarized and fed back to practitioners to encourage practice change, is routinely and increasingly employed in many clinical contexts. The most recent Cochrane review on the effectiveness of A&F interventions includes 140 trials [1] and shows that such interventions yield modest (median adjusted risk difference of 4.3% absolute increase) but variable (interquartile range of 0.5 to 16%) improvements in clinical practice. Despite so much collective experience, a cumulative analysis of estimates of effect by year indicated that effects’ sizes plateaued sometime around 2003 [2], suggesting our efforts to design effective A&F interventions are not improving.

We propose that a key factor impeding progression towards more effective A&F healthcare interventions has been a lack of theoretical understanding of the mechanisms underlying these interventions. We have shown that theory is rarely invoked in the design of health care A&F interventions; less than 10% of A&F interventions report any theory guiding design of the intervention [3]. Instead, the majority of current A&F interventions appear to be guided by intuitive, non-theoretical ideas about what might work [4]. Without the application of theory, one cannot predict whether a successful intervention will generalize, learn much from failed interventions, or successfully tailor interventions to a new context [5, 6].

Attempts to apply theory to A&F have focused on individual theories from health and social psychology [7] and organizational science [8]. For example, a systematic review using constructs informed by the Feedback Intervention Theory from organizational psychology [8] identified specific constructs (e.g., frequency of feedback, patient-specific feedback) that are likely related to A&F effectiveness. However, there is a much broader range of theories that may suggest explicit, testable hypotheses about how to optimize A&F interventions [9]. For example, using social interaction to increase what can be learned from A&F [10] could provide a rich source of theory-informed concepts regarding how to design more effective A&F. Our team has argued that when seeking to identify theoretical mechanisms underlying complex interventions, existing theories describing specific sub-components of these interventions may suggest ways to improve the overall intervention [11]. This idea opens up a much wider range of theoretical perspectives than has currently been applied to healthcare A&F.

We propose that the literature on healthcare A&F interventions will be advanced by consideration of ideas from a broad range of relevant theoretical traditions. Specific methods for incorporating theory from many disciplines are limited, making it necessary to generate novel approaches. Our approach involved identifying and interviewing experts from specific, a priori defined theoretical traditions, providing examples of healthcare A&F, and eliciting explicit, theory-informed hypotheses about how that A&F could be improved. The objective of this study was to use this approach to develop a broad list of testable, theory-informed hypotheses about how to improve A&F interventions.

## Methods

Our study used semi-structured, in-depth interviews with theory experts to identify specific hypotheses and a thematic analysis to organize the resulting hypotheses into themes. Ethics approval was obtained from the Ottawa Health Sciences Network Research Ethics Board.

### Participants

Using purposive sampling, a preliminary list of theory experts from a priori defined relevant fields (i.e., cognitive psychology, social or health psychology, education, medical decision-making, industrial/organizational psychology, and economics) was developed by the study team. The list was developed based on the research team’s respective knowledge of theorists whose publication history and impact made it clear they would provide a useful perspective on how to improve A&F interventions. In order to qualify as a theory expert, the potential participant had to have demonstrated expertise in one or more relevant theories. The goal was to attempt coverage across a broad range of fields deemed by the research team to have relevance to the study or use of A&F. Interviewed participants were asked to suggest others whose input they judged would be valuable based on the interview experience (i.e., snowball sampling). We re-evaluated our list of potential participants on a number of occasions to ensure that our sample included, to our knowledge, the most relevant theorists and a broad sample from a range of fields. Participants were given a $200 CAD honorarium for their participation.

### Describing our sample and related theoretical expertise

We categorized each participant into a field based on their self-described primary area of expertise or discipline. For the purposes of describing field coverage, experts with extensive expertise in two of the a priori defined fields were coded twice. We also created a summary of the theories and/or theoretical concepts described by the participants as informing their work through analysis of (1) their answer to the first interview question specific to their area of theoretical expertise and (2) additional theories and/or theoretical concepts discussed during the course of the interview.

### The interview guide and interview

Experts were sent materials prior to the interview session, including the interview questions, a summary description of four published A&F randomized trials, and the relevant trial publications [12–15]. The four target examples were intended to represent a range of common healthcare A&F interventions and differed in many ways (i.e., whether the A&F was group or individual, whether the A&F was given to the target for behavior change, what the A&F was about, the use of target goals or benchmarks, key educational messages, and the frequency of the A&F). Additional file 1 includes a description of the four A&F interventions. Interviewees were asked to read the material in preparation for the interview.

The interview guide (see Additional file 1) was pilot tested in four interviews to establish the appropriateness, flow, and robustness of the guide. All interviews were conducted by three members of the team (HLC, JCB, KC). Interviews were conducted by telephone, audio-recorded, and lasted between 60 and 90 min. Interviews covered 2–4 of the A&F examples, depending on how the conversations went. We planned to cease sampling once we achieved coverage of our a priori defined fields and saturation for theme development (i.e., new interviews generally fit within the current thematic coding frame with no new themes identified).

The interview consisted of three main tasks. The first asked the expert to describe their theoretical expertise and the theories that guide their work. This allowed the participant to review their own foci prior to exploring the A&F interventions and oriented the interviewers to the participant’s specific theoretical expertise, jargon, and approach to the concept of A&F. The second task involved one interviewer (HLC) explaining, one by one, and in detail, up to four A&F interventions. For each, the participant was asked to provide their initial open-ended reactions to each A&F intervention. They were then asked to comment on aspects they liked or disliked about each intervention and how they would go about improving it. In doing so, they were encouraged to describe their input in terms of specific theory-based and specified hypotheses for more effective A&F as much as possible (as opposed to intuitive ideas on designing better A&F). Interviewers asked clarifying questions when needed and engaged in discussion aimed at understanding the hypotheses proposed and the theory behind them. Interviewers sought to identify what theoretical perspective led to each hypothesis. When the discussion did not make it clear, this link was further sought as part of the member checking process. The third and final task was to ask the participant if they had any additional thoughts on how best to design A&F interventions that were not discussed during the review of the examples. This task was intended to facilitate discussion of a broader range of hypotheses related to A&F effectiveness beyond those invoked by the four specific examples.

### Member checking the hypotheses

Following each interview, and using the audio-recording and notes as a guide, one member of the research team (KC) developed a draft member checking document outlining the testable hypotheses described in the interview. This document was used to confirm that the research team correctly understood the expert’s perspective. A table was developed that summarized the concept or idea behind the hypothesis, the specific hypothesis, and where possible, relevant mechanisms of action, mediators, outcomes, contextual factors and theories guiding the hypothesis. The draft was iteratively reviewed and modified by two other members of the research team (HLC, JCB) until all three agreed as to accuracy and completeness. The theory expert was then asked to review and use track changes to modify and add additional detail or clarity to the final document.

### Theme generation

In order to organize the hypotheses from all final interview documents, we used a process similar to the constant comparative method of data analysis (open coding) used in qualitative research [16]. Hypotheses were independently assigned to themes in an iterative process by 3 coders (JCB, HLC, KC). This was done in blocks of 50 or 100 randomly chosen hypotheses. A consensus meeting was held to review proposed themes and develop an initial coding frame. After each block, we repeated the process and modified the coding frame with new themes as needed. Our focus was to get a clear understanding of the full range of hypotheses, not to keep the themes to a minimum. Prior to finalizing the themes, we identified and removed the hypotheses that were identical or duplicated, resulting in a total number of unique hypotheses. If hypotheses were similar, but seemed conceptually different for any reason, we did not designate the hypotheses as duplicate. This process was conducted by two team members separately (HLC, KC), followed by a consensus discussion for any discrepancies. The final thematic structure was confirmed by a fourth member of the team (KWE). The final task involved grouping the 30 themes into general categories. This was conducted by three members of the team separately (HLC, KC, KWE) followed by a consensus discussion and confirmation by a fourth member of the team (JCB).

## Results

We approached 47 theorists over a 1-year period. Twenty-eight (60%) agreed to participate and underwent a full interview. Five refused to participate; three were too busy, and two expressed lack of expertise. An additional 14 did not respond. Table 1 describes the disciplines or fields of the participants. Eighteen of the 28 participants were from the USA, five were from Canada, and the remaining five were from various countries in Europe. The discipline or field with the most participants was cognitive psychology (*n* = 9), and the least represented was human factors (*n* = 2).

**Table 1:** Expertise of participants by discipline or field and total, *n* = 28

Discipline or field	Total
Cognitive psychology	9
Education	8
Medical decision-making	7
Industrial organization or management	6
Social or health psychology	5
Medical education	5
Economics	3
Human factors	2

17 participants were categorized into two disciplines or fields

Table 2 describes the range of expertise described by the participants as informing their work. Several participants cited expertise in Goal Setting Theory [17, 18], Control Theory [19], Self-Regulation Theory [20], Self-Efficacy [21], and various behavior change and learning theories. Together, there were over 100 different areas of expertise provided by the participants.

**Table 2:** Range of self-described expertise and other areas of expertise by participant

Expert	Self-described expertise	Other concepts/areas of expertise referred to during interview
1.	Brunswikian psychology, diagnostic judgments of physicians, use of vignettes containing clinical cues	Lens modeling, evidence-based medicine, behavior change theory, face validity
2.	Behavior decision theory, methodological theory of information integration—what cues people pay attention to	Diffusion of responsibility, norm theory, SMART goals, loss aversion theory, scale compatibility, habituation theory, spacing effects
3.	Human factors, health communication and decision making, gestalt principles, information science	Theories of attention, international design standards, prospect theory, loss aversion, cognitive load, constructivist learning theory, intrinsic/extrinsic motivation
4.	Self-assessment, behavior change, comparison models	Guided reflection, learner centered agenda, teacher directed agenda
5.	Diagnostic reasoning of physicians, dual process models, information distortion (gestalt)	Learning theories, theory of planned behavior, extrinsic motivation
6.	Human factors engineering, iterative design	Theory of planned behavior, theories of operant conditioning, law of effect
7.	Cognitive psychology, judgment decision making framework, information processing, linguistics	Tufte theory, incentives, Lake Woebegone effect in social psychology
8.	Applied work in medical decision making, hindsight bias	Fast and frugal heuristics
9.	Cognitive psychology—how people reason, formulate judgments, and make decisions	
10.	Personality, social, and health psychology, principles of feedback control	Self-regulation of behavior
11.	Cognitive psychology, learning, memory, lab research on feedback	
12.	Behavioral economics, psychology, rational choice	Individual limitations, motivations, ego, mastery, social comparison
13.	Organizational psychology, feedback research, feedback seeking behavior, feedback environment framework, information processing, achievement goal theory, personality	Self-motives framework, self-enhancement theory, self-determination theory, motivation, dual process models, serial position curve/memory
14.	Cognitive psychology, measurement, assessment, formative feedback, constructivism, active learning theories	Cognitive load, growth mindset work (Dweck), goal setting theory, display of quantitative information (Tufte), graph design
15.	Social psychology, attribution/dissonance theory, prospect theory, conflict and dispute resolution, study of influence	Lewinian channel factor identification, self-perception theory, social norms theory, nudge theory (Thaler and Sunstein), motivation, Prospect Theory
16.	Methodology, resource management principal	
17.	Bjork’s desirable difficulties	
18.	Psychology, dual processes, affect and emotion, numeracy and aging	
19.	Goal setting theory	Cognitive load
20.	Social psychology, health communication strategies or health decision making and health behavior change, social cognitive theory, theory of planned behavior, adoption process model, social comparison theory, classic theories of attitude and behavior	Study of influence
21.	Control theory, self-regulation theory, goal setting theory, self-efficacy	Reinforcement theory, partial reinforcement theory
22.	Industrial organizational psychology, work motivation, team performance, feedback from the standpoint of individual behavior	Self-regulation, control theory, goals and actions, subjective expected utility theory (theory of reasoned action, expectancy theory)
23.	Educational theory, learning theories, constructivism, socio-cultural learning theories	Reflective learning, motivation, peer learning, communities of practice, social learning, peer scaffolding, role modeling
24.	Applying psychological principals in clinical practice, sociological learning theory, sociocultural theory, feedback interventions	Discourse theory, activity theory, complexity theory, achievement motivation theory
25.	Psychology, economics, ethics, patient physician communication, treatment decision making	
26.	Education research, feedback in education, social cultural theory	
27.	Industrial organizational psychology, Power’s control theory (self-regulation theory), Carver and Scheier’s social cognitive theory	Gain theory, implementation intentions
28.	Education, constructivist approach, basic notions of social psychology, multisource feedback or feedback to students from supervisors (Ross and Nesbett), social cultural theory (Vigotsky), humanist theory (Carl Rogers)	Theories of behavior change, self-regulation, feedback intervention theory (Kluger and Denisi), motivation theories, informed self-assessment

### Hypotheses generated

The 28 interviews yielded a total of 389 hypotheses. After duplicates were removed, 313 unique hypotheses remained. These hypotheses were organized into a coding framework with 30 themes (Table 3) across five categories. The complete list of all 313 unique hypotheses, organized according to theme and category, can be found in Additional file 2.

**Table 3:** Summary of hypotheses by theme and with examples

Themes(*N* = 30)	# of hypotheses (*N* = 313)	Example hypotheses A&F/A&F interventions will be more effective…
Related to the recipient		
1. Trust/credibility	14	If it is perceived to be without conflict of interest; when recommendations related to the A&F are based on good quality evidence
2. Motivation/intention	13	If it is accompanied with positive reinforcement to those who have improved their performance; when accompanied by incentive
3. Recipient characteristics	9	For those with a mastery goal orientation if it involves comparison to others
4. Recipient priorities	9	When targeted at behaviors that the target feels is important to their professional roles/responsibilities
5. Attack on self-identity	7	When measures are used to prevent a defensive response (providing other “reassuring” messages as well)
6. Attract/maintain attention	6	If they engage the target’s attention
7. Self-efficacy/control	5	If the behavior is under the control of the recipient
Related to the behavior		
8. Remove barriers	11	If they address barriers to change in behavior
9. About aspects of behavior	7	For behaviors that are easy compared to those that are harder to do
10. Decision processes or conceptual model	4	If designed with a clear understanding of the decision making process underlying the behavior to be changed
Related to the content of the A&F		
11. Cognitive load	33	If as few graphs as possible are presented; without unnecessary depth elements; if the graphical representations are clearly and consistently labeled; when color changes are purposeful and convey meaning; when presenting absolute numbers as opposed to percentages; when graphical clutter is removed; when focused on a few, most important behaviors
12. Comparisons	26	When the benchmark comparison is justified to be a reasonable standard; when a comparator is provided; when multiple individual practice data is presented along with the recipient’s data; if it involves a comparison to the self; if the comparator is specific to the recipient’s own context/practice.
13. Action plans/coping strategies	19	If clear direction on how to change behavior is provided
14. Feedback specificity	16	If individual level provider data is provided; if patient-specific information is provided; if it is as specific as possible
15. Goal setting	16	If it is accompanied by a goal that is specific
16. Justify need for behavior change	10	If accompanied by evidence supporting the behavior change
17. Cognitive influences	7	If emphasis is on what needs to be achieved (loss framing) as opposed to what was achieved (gain framing).
18. Nature of the data	6	If graphical representation displays the variability of data in order to indicate the error or uncertainty
19. Guide reflection	6	If it involves a personal reflection component
20. Improving memory	6	If the reminder messages are presented in real time/point of care; if incorporates an emotional message underlining the desired behavior
Related to the delivery of the A&F		
21. A&F timing	20	If individual change data over time is provided; when presented multiple times; when presented at the time of decision making
22. Social engagement	17	If they involve engaging recipients in social discussion about the A&F
23. Knowledge/learning	13	If it creates opportunities to learn
24. User-guided experience	6	When complex information is scaffolded to allow a recipient to get more information if and when they want
25. In-person A&F	2	When provided with human contact
26. Responding to A&F providers	2	If they allow the recipient an opportunity to indicate why a recommended action was not taken.
Other		
27. Opportunity costs	7	When there are few costs to change behavior
28. Environment	4	If the environment encourages the desired behavior as the default.
29. Development process involvement	2	When recipients have been involved in the design of the A&F
30. Single hypotheses	10	If they imply some kind of extended commitment; if the recipient generates a response immediately prior to receiving the A&F; if the goal is made public; if it is provided to the intended target for behavior change; it includes multiple modes of information (e.g., pictures and text)

### Related to the recipient (*n* = 63 hypotheses in seven themes)

The hypotheses and themes in this category pertain to the reaction or perspective of the recipient of the A&F. The largest theme in this category was *trustworthiness/credibility*, which contained 14 hypotheses all outlining the importance of considering the degree to which a recipient trusts the source of and/or data in the A&F (e.g., A&F will be more effective if it is perceived to be without conflict of interest, when recommendations related to the A&F are based on good quality evidence). Thirteen hypotheses related to *motivation/intention* issues of the recipient, such as using positive reinforcement (e.g., A&F will be more effective over time if it is accompanied with positive reinforcement to those who have improved their performance). The theme *recipient characteristics* contained nine hypotheses, all related to how attributes of the recipient of the A&F should inform the A&F design (e.g., A&F will be more effective for those with a mastery goal orientation if it involves comparison to others). Nine hypotheses outlined the importance of ensuring an understanding of *recipient priorities* (e.g., A&F will be more effective when targeted at behaviors that the recipient feels are important to their professional roles/responsibilities), and seven hypotheses were in the theme *attack on self-identity* and described how A&F needs to ensure that defensive reactions do not take place (e.g., A&F will be more effective when measures are used to prevent a defensive response—providing other “reassuring” messages as well). The last two themes (six and five hypotheses respectively) contained hypotheses related to how best to *attract and maintain attention* of the recipient (e.g., A&F will be more effective if they engage the target’s attention), and how to design A&F to maintain *self-efficacy/control* (e.g., A&F will be more effective if the behavior is under the control of the recipient).

### Related to the behavior (*n* = 22 hypotheses in three themes)

All of the hypotheses and themes in this category were focused on the behavior that the A&F intervention was meant to change. The largest theme was *remove barriers* which included 11 hypotheses that encouraged an understanding of the specific barriers to the behavior (e.g., A&F will be more effective if based on a barriers analysis). There were seven hypotheses in the theme *about aspects of the behavior* that outlined conditions related to the behavior itself (e.g., A&F will be more effective for behaviors that are easy compared to those that are harder to do). The last theme, *decision processes or conceptual model*, pertained to ensuring a good understanding of behavioral decision-making (e.g., A&F will be more effective if designed with a clear understanding of the decision making process underlying the behavior to be changed).

### Related to the content of the A&F (*n* = 145 hypotheses in ten themes)

All of the hypotheses and themes in this category were focused on the content included in the A&F. The theme with the most hypotheses in this category was *cognitive load,* which contained 33 hypotheses all related to reducing the amount of mental effort required to mentally process the A&F. It included hypotheses related to overall simplicity (e.g., A&F will be more effective if as few graphs as possible are presented), the display of the A&F (e.g., A&F will be more effective when color changes are purposeful and convey meaning), and the content of the A&F (e.g., A&F will be more effective when focused on the few, most important behaviors). Twenty-six hypotheses focused on *comparisons* including the use of benchmarks as comparisons in the A&F (e.g., A&F will be more effective when the benchmark comparison is justified to be a reasonable standard), comparisons in general, social comparisons (e.g., A&F will be more effective when multiple individual physician practice data is presented along with the recipients’ data), comparisons to the self, and the specificity of the comparison. Nineteen hypotheses related to *enabling action plans/coping strategies* (e.g., A&F will be more effective if clear direction is provided on how to change behavior). *A&F specificity* included hypotheses related to A&F being specific to the individual, being patient specific, or around the ideal level of specificity (e.g., A&F will be more effective if it is as specific as possible). The positive effect of *goal setting* within A&F was addressed in 16 hypotheses (e.g., A&F is will be more effective if accompanied by a goal that is specific). The remaining themes included hypotheses related to ensuring that the A&F *justifies the need for behavior change*, other *cognitive influences*, the *nature of the data* presented, designing the content such that it guides the recipient (*guide reflection*), and *improving memory* by using reminders.

### Related to the delivery of the A&F (*n* = 60 hypotheses in six themes)

All of the hypotheses and themes in this category were focused on the processes used when delivering the A&F, regardless of the content of the A&F. The largest theme in this category included 20 hypotheses related to *A&F timing*. This included hypotheses about providing A&F over time (e.g., A&F will be more effective if individual change data over time is provided), multiple times, and other timing-related issues (e.g., A&F will be more effective when presented at the time of decision making). The theme of *social engagement* had 17 hypotheses related to engaging recipients in social discussion about the A&F (e.g., A&F interventions will be more effective when they incorporate facilitated social discussions about the A&F). Thirteen hypotheses focused on knowledge/learning (e.g., A&F that creates opportunities to learn will be more effective). The remaining themes included hypotheses related to allowing the recipient to control how they access the A&F (*user-guided experience*), delivering the A&F in person (*in-person feedback*), and delivering the A&F such that the recipient is asked to respond to the A&F (*responding to feedback providers*)*.*

### Other (*n* = 23 hypotheses in four themes)

This category includes three themes that did not relate to the four categories above, as well as a grouping of ten single hypotheses that did not relate to any theme. There were seven hypotheses that outlined the importance of considering the *opportunity costs* of the A&F (e.g., A&F is more effective when there are few costs to change behavior), four hypotheses that related to the *environment* (e.g., A&F is more effective if the environment encourages the desired behavior as the default), and two hypotheses related to *development process involvement*, or including the recipients in the design of the A&F (e.g., A&F is more effective when recipients have been involved in the design of the A&F). A notable single hypothesis was that A&F will be more effective if made publicly available.

## Discussion

In an effort to broaden the range of theoretical perspectives to apply to health care A&F, we successfully interviewed 28 theory experts from a broad set of theoretical perspectives and fields and created a list of testable, theory-informed hypotheses about how healthcare A&F interventions might be improved. We developed a list of 313 unique hypotheses in 30 themes. To our knowledge, this is the first explicit effort to bring theory from many different relevant disciplines to the problem of optimizing health care A&F interventions. Our approach was successful in yielding new hypotheses that are not currently captured in existing A&F theories and that, to our knowledge, have not been tested in evaluation studies of A&F [1]. The hypotheses and/or themes presented in this paper will form the basis of a future prioritization exercise designed to support a coherent, theory-guided research agenda for optimizing A&F as an implementation intervention. Developing this agenda will be the next step of this work.

Currently, both intervention development and evaluation of A&F interventions are driven primarily by the intuitions of individual investigators [3]. This work provides an initial step towards a more theory-guided science of A&F development and evaluation. This sort of organized approach to evaluation has been highlighted as an essential future research direction for this field [2]. We expect this work to not only help prioritize research directions for the field but also encourage ambitious large-scale trials comparing multiple approaches to A&F [22] and to assist A&F laboratories tasked with exploring and designing innovative interventions involving A&F [23].

The number of potential hypotheses identified and the range of theories and theoretical concepts discussed underscores the complexity and number of potential mechanisms underlying effective A&F. We found that constructs from well-known theories specific to A&F were well represented in our hypotheses. For example, constructs from the Feedback Intervention Theory [8] such as feedback timing (i.e., the more frequent the better), the importance of goal setting, and the role of personality on the reaction to feedback were clearly represented in our list. Constructs from Hysong’s Model of Actionable Feedback [24] were also represented (e.g., feedback needs to be timely, individualized, non-punitive, and customizable). Importantly, however, many of the hypotheses generated (e.g., related to social engagement, trustworthiness/credibility, removing barriers, justifying need for behavior change, nature of the data, environment) are not represented in A&F-specific theories and instead stem from theories that might be seen as overlapping with A&F or target components of A&F, rather than describing A&F itself. Consider the following:

- A&F will be more effective if noun descriptors rather than verbs are used in messaging—‘do not be an over prescriber’ versus ‘please prescribe less’.
- A&F will be more effective if it incorporates a gaming approach.
- A&F will be more effective if information about opportunity costs is included; A&F will be more effective when recipients have been involved in the design of the A&F.
- A&F interventions will be more effective if they involve engaging recipients in social discussion about the A&F.

None of these hypotheses are specified as part of existing theories of A&F, but nevertheless suggest potentially innovative ways to improve this class of interventions. These findings suggest that there may be more to be learned about the A&F process if we allow ourselves to incorporate constructs and mechanisms from other theories [11].

The initial focus of this work was to generate testable hypotheses that were clearly and closely tied to the specific theories in which our participants were most expert. Despite our efforts in both the interviews and in the member checking process, participants often had considerable difficulty and/or showed reluctance to explicitly tie hypotheses to specific theories. The theory experts were probed about their specific theoretical orientation and were told to focus on hypotheses with a theoretical basis (as opposed to intuitive ideas), yet it became clear that the hypotheses being generated varied substantially in terms of how clearly they could be mapped onto a specific theory. For example, hypotheses related to goal setting could easily be mapped to theory [17]; in contrast, many hypotheses related to cognitive load (e.g., remove graphical clutter, label consistently and clearly) were less likely to be ascribed to a specific theory by our experts. In general, while we believe most experts adhered to our instructions and tried to generate theory-based hypotheses, it is possible that a subset of our hypotheses are better described as the intuitions based on the experience of theory experts [25], rather than hypotheses clearly predicted by theory. Regardless, we see this work as being a possible and potentially more direct route towards guiding the A&F research community towards better interventions than the current serendipity-driven and intuition-based approach [4].

With such a vast list, we felt obliged to organize into themes and categories, yet without a definitive taxonomy of A&F, we proposed only a simple, descriptive structure based on team consensus. Our efforts were designed to organize the themes into a manageable number of categories and not to propose a framework with any implied structure of importance or relevance outside of the summary. Our category scheme (relevant to *the recipient, the behavior, the content*, and *the delivery* of the A&F) is one way to frame important A&F elements; others have organized them differently [2, 4, 24, 26, 27]. A definitive taxonomy of A&F interventions would help standardize how A&F interventions are designed, described, and reported.

A number of additional challenges of this work warrant consideration. First, these interviews were extremely labor intensive and challenging, often involving unfamiliar jargon; it is therefore likely that some of the subtleties of the various concepts discussed were lost, despite an extensive and iterative member checking process. We feel that part of the innovation of this approach was the focus of the interviews on testable hypotheses, which facilitated in-depth discussion between interviewers and interviewees despite quite different expertise. Another possible limitation stems from the 4 specific A&F interventions that were the focus of discussions; different hypotheses may have been generated had we chosen different A&F examples. Indeed, most of our discussions started out with the participant talking about display issues specific to the individual example. We sought to overcome this potential demand characteristic by specifically asking for thoughts on A&F in general as opposed to the examples, but it is very possible that the examples directed interviewee attention towards specific issues. This is one of the reasons why we believe it would be an error to equate frequency with which a hypothesis was mentioned with its potential importance or priority for study. Again, however, we see this design choice as part of the innovation of our approach, as these examples facilitated in depth discussion between people of very different expertise. Finally, we cannot guarantee that our sample covered all relevant disciplines, theoretical perspectives, and geographical areas (i.e., the sample was exclusively North American and European). This is, however, the largest compilation of A&F-relevant hypotheses to date. The approach provided an extensive list of testable hypotheses that would have been far more difficult (or impossible) to achieve through other approaches (e.g., literature review), and that includes hypotheses novel to the healthcare A&F literature.

## Conclusion

The development of the scientific basis of A&F in healthcare appears to have stagnated; we are not developing more effective A&F interventions than we were 20 years ago. We developed a methodology that would allow this area to be informed by a much wider range of theoretical work than was possible previously. Our list of theory-informed hypotheses will be an important foundation for moving this literature forward, enabling prioritization exercises, head-to-head trials where the arms are informed by theory and not just investigator intuition, more comprehensive theoretical descriptions of A&F processes, and ultimately more consistently effective A&F interventions. With such a list, the field will be better positioned to systematically guide the continued evolution of this important intervention.

## Additional files

Additional file 1: The interview guide (or supplemental file with the appendix below in the paper). (DOCX 369 kb)

Additional file 2: All 313 hypotheses organized in 30 themes. (DOCX 37 kb)

## Abbreviation

A&F: Audit and feedback
