# Supplementary material for: Advancing the literature on designing audit and feedback interventions: identifying theory-informed hypotheses
Source: Implement Sci. 2017 Sep 29;12:117. doi: 10.1186/s13012-017-0646-0 (PMC5622490; doi:10.1186/s13012-017-0646-0)
Supplement: Supplementary file 1 — The interview guide (or supplemental file with the appendix below in the paper). (DOCX 369 kb) [file 13012_2017_646_MOESM1_ESM.docx]

Additional file 1: The Interview Guide with Theory Experts

Thank you very much for agreeing to help us with this study. As you may know, providing health care practitioners with new research information in a way that will encourage improvements in health care is challenging. Audit and Feedback (A&F), a type of intervention where current behaviour of providers is assessed and fed back to them, often for the purpose of encouraging that they change their behaviour to be more in line with best practices, is considered one of the most promising methods for achieving such improvements, and has been shown in systematic reviews to be effective in some situations (Ivers et al: Audit and feedback: effects on professional practice and healthcare outcomes. (Cochrane Database of Systematic Reviews 2012, Issue 6: CD 000259).

What has been lacking in this literature is a theoretical understanding of what leads to effective or ineffective A&F interventions. This CIHR-funded study is designed to assess whether theories and theory-motivated hypotheses from other literatures can help make more effective A&F interventions.

What I’d like to do today is to present you with some target A&F intervention studies and reflect on what theories or theory-motivated hypotheses you think should be considered as we try study how to improve this kind of intervention.

To start off, can you tell us about **your theoretical expertise?** What are the main theories that guide your work?

Now, let’s move on **to our feedback examples**. We are going to start with one of three examples, first drawing your attention to some key aspects of the feedback. These are “real world” examples of audit and feedback intervention studies; as such, we can often only provide what information was provided in the publication, but we’ll do our best to answer any questions you have.

We’ll then ask you to work through the example and think about it a bit, particularly about how you might do things differently. We‘ll get you to explain the A&F back to us as an approach to ensure that you have understood the feedback and are ready to discuss it.

You might find it helpful to have a piece of paper handy to jot down any ideas you might have.

Ready? Let’s begin….

**Audit & Feedback Example #1: DRAM Trial**

*Reference:* Effect of enhanced feedback and brief educational reminder messages on laboratory test requesting in primary care: a cluster randomised trial; Lancet 2006, 367:1990-96. Thomas RE., Croal BL., Ramsay C., Eccles M., Grimshaw J.

This study evaluated the effectiveness of audit and feedback (around ordering rates for various lab tests) enhanced with educational reminder messages describing what inappropriate use would look like for **nine laboratory tests** commonly used in primary care. The study involved 85 family physician practices (~370 family practitioners) in Scotland. Test ordering rates for the practice group (not the individual physicians), **enhanced with the educational messages** were sent to individual family physicians on four occasions.

Some key aspects of the Feedback (you might want to look at the graph on the next page as we go through these points):

- Feedback provided was at the level of the practice (not the individual)
- A 6- page colour booklet was sent to individual family physicians
- 12 graphs in total; 3 graphs summarized the laboratory test areas as a whole; 9 graphs summarized the specific lab tests. Two of the graphs are presented here.
- The graphs displayed the practices request rate for the lab test for every 10,000 patients for a 6-month period of time.
- Graphs compared the average of their practice group (red line) to the regional average (blue line)
- Educational messages describing inappropriate use of the specific lab test were presented alongside the graphs
- Feedback was sent four times over the course of 1 year
- Context: There is an over ordering of laboratory tests in primary care, thus this intervention was about trying to reduce the number of unnecessary test requesting.

**Please see next page for an example of the feedback form**


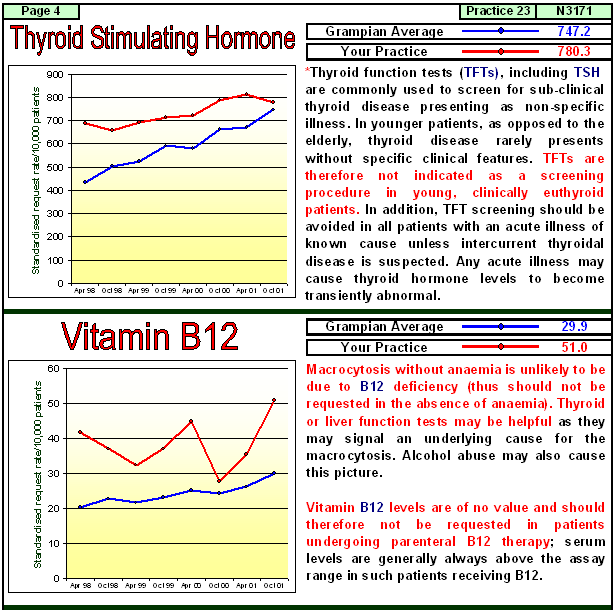


**Things to think about as you reflect on the A&F intervention**

- Given your theoretical expertise, how do you think the intervention would work and/or how would you go about improving an intervention like this?

Let’s focus on a particular hypothesis your theory suggests…

- What would that be? Can you frame a clear **hypothesis** for us?
- What is the **theoretical mechanism of action**? (i.e. what makes this effective?)
- On what **outcomes** would this hypothesis show its effects?
- Are there any **contextual factors** that would affect the hypothesis? (i.e. for whom and under what circumstances would this be effective?)
- Can you identify a specific **theory or model** that motivates this hypothesis?
- Are there any **specific readings** you would point us to about these ideas?

*What else?*

- **Reminder**: The goal of this exercise is *to* generate ***theory- informed hypotheses*** (as opposed to just general ideas) that you think should be considered in improving this kind of intervention.

**Audit & Feedback Example #2: Tobacco Cessation Quit Line**

*Reference:* Practice-based referrals to a tobacco cessation quit line: assessing the impact of comparative feedback vs general reminders. *Annals of Family Medicine* 2007: 5(2): 135-142. Wadland WC., Holtrop JS., Weismantel D., Pathak PK., Fadel H., Powell J.

This study assessed the impact of **audit and feedback vs general reminders** as approaches to increase primary care referrals to a tobacco quit line service. The study involved 308 physicians from 87 primary care practices in Michigan. Physicians were given feedback on their individual and practice group referral rates over an 18 month period.

Some key aspects of the Feedback (you might want to look at the graph on the next page as we go through these points):

- Feedback was on the performance of both the individual physician and the practice group
- Individual-level feedback was compared to the physician’s practice average
- And it was compared to an average of all physician practices in the study
- Feedback was presented in the context of a goal or benchmark, defined as the average of the top performing 10% of study physicians
- Individual physicians were sent a 1-page report showing 2 graphs
- Graph 1: Indicates how close the doctor is to the benchmark, explained as a % achieved towards benchmark, and compared to the doctors practice group and an average of everyone in the study
- Graph 2: The individual physician’s actual number of referrals for each quarter
- Key messages presented below the graphs indicated individual number of referrals and how many more referrals were needed to reach the benchmark
- Feedback was mailed to physicians every 3 months for a total of 6 mailings
- Context: To increase the uptake of clinician referrals to a smoking cessation quit line.

**Please see next page for an example of the feedback form**


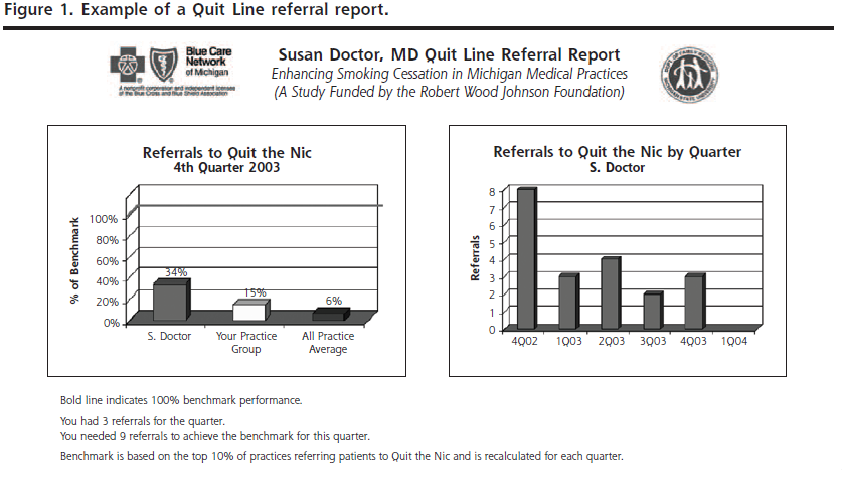
**Audit & Feedback Example #3: Effective Cardiac Treatment**

**Things to think about as you reflect on the A&F intervention**

- Given your theoretical expertise, how do you think the intervention would work and/or how would you go about improving an intervention like this?

Let’s focus on a particular hypothesis your theory suggests…

- What would that be? Can you frame a clear **hypothesis** for us?
- What is the **theoretical mechanism of action**? (i.e. what makes this effective?)
- On what **outcomes** would this hypothesis show its effects?
- Are there any **contextual factors** that would affect the hypothesis? (i.e. for whom and under what circumstances would this be effective?)
- Can you identify a specific **theory or model** that motivates this hypothesis?
- Are there any **specific readings** you would point us to about these ideas?

*What else?*

- **Reminder**: The goal of this exercise is *to* generate ***theory- informed hypotheses*** (as opposed to just general ideas) that you think should be considered in improving this kind of intervention.

*Reference*: Administrative data feedback for effective cardiac treatment; JAMA 2005, 294: 309-317. Beck CA., Hugues R., Tu JV., Pilote L.

This study evaluated whether audit and feedback based on hospital and prescription administrative databases is effective for **improving quality of care for acute myocardial infarction** (AMI). Quality indicators reflecting patient outcomes for acute myocardial infarction (AMI) care were sent to 77 acute care hospitals in Quebec.

**Some key aspects of the Feedback** (you might want to look at the graph on the next page as we go through these points):

- Feedback was provided in the form of a hospital report card
- Feedback was mailed to directors of services (not the physicians who treated the patients) with the encouragement for them to share with those who treat the patients
- Educational materials to support this sharing were provided
- Hospital-specific feedback was compared to the average of all teaching hospitals
- And it was compared to all hospitals in the province of Quebec
- Feedback included 2 histograms for each of the 12 different aspects of care for acute myocardial infarction (24 histograms in total)
- Histogram 1: The percent of patients receiving appropriate care with the 2 comparators
- Histogram 2: Shows the distribution of hospital performance for that aspect of care
- A recommended target rate was included, but it’s not clear what it was based on
- Feedback was sent once; based on data that was summarized from the previous year
- Context: Many patients are not receiving the recommended treatments for AMI, thus this intervention was about increasing the degree to which quality of care indicators are met for cardiac treatment.

**Please see next page for an example of the feedback form**


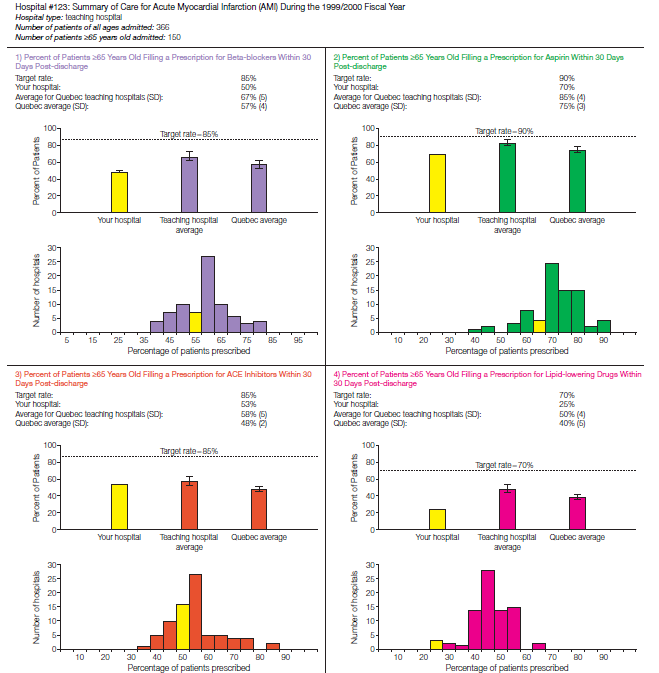


**Things to think about as you reflect on the A&F intervention**

- Given your theoretical expertise, how do you think the intervention would work and/or how would you go about improving an intervention like this?

Let’s focus on a particular hypothesis your theory suggests…

- What would that be? Can you frame a clear **hypothesis** for us?
- What is the **theoretical mechanism of action**? (i.e. what makes this effective?)
- On what **outcomes** would this hypothesis show its effects?
- Are there any **contextual factors** that would affect the hypothesis? (i.e. for whom and under what circumstances would this be effective?)
- Can you identify a specific **theory or model** that motivates this hypothesis?
- Are there any **specific readings** you would point us to about these ideas?

*What else?*

- **Reminder**: The goal of this exercise is *to* generate ***theory- informed hypotheses*** (as opposed to just general ideas) that you think should be considered in improving this kind of intervention.

**Audit & Feedback Example #4: Preventive Care**

*Reference:* Delayed Feedback of Physician Performance Versus Immediate Reminders to Perform Preventive Care: Effects on Physician Compliance; Medical Care 1986, 24(8): 659-666. Tierney WM., Hui SL., McDonald CJ.

This study evaluated the effectiveness of audit and feedback and reminders as approaches to increase compliance with preventive care actions (e.g fecal occult blood testing, oral calcium supplementation). The study involved 135 internal medicine interns and residents in Indianapolis. Thirteen preventative care actions were randomly divided into two feedback groups; half of the interns/residents were given monthly feedback for Group A actions, and the other half for Group B actions. Thus each feedback group acted as a control for the other. Within feedback groups, individuals were randomly assigned also to receive immediate reminders (given at time of patient visits) for either Group A or Group B actions.

Some key aspects of the Feedback (you might want to look at the sample feedback report on the next page as we go through these points):

- Feedback reports provided to the individual physician about their own patients
- Each month, records were searched for patients who had visited a physician in the clinic and had an indication for, but did not receive, one or more of the preventative care actions.
- The feedback reports sent to the physicians identified each patient by NAME, their age, the dates of the last and next scheduled appointments, the action that was NOT taken, and information that made the patient eligible for the action
- Each physician was required to indicate on the feedback report what action should be taken from five options: rescheduling the patient sooner, marking the form to suggest the physician perform the preventative care on the next visit, indicating the protocol was not applicable for that specific patient, stopping the reminder for that patient, or pulling the patients chart for review. The example on the next page is this feedback report.
- Feedback was sent monthly over the course of 7 months. The number of patients included in the feedback reports varied by how many patients had not received the preventative care actions
- Reminders for some patients were generated the night before the scheduled appointment and placed in the patients’ charts. Reminders identified the patient, recommended relevant preventative care actions, data that made the patient eligible for the action, and supporting references.
- Context: Physicians do not consistently order preventive care measures for their eligible patients, thus this intervention was about trying to increase compliance with preventive care guidelines.

**Please see next page for an example of the feedback form**


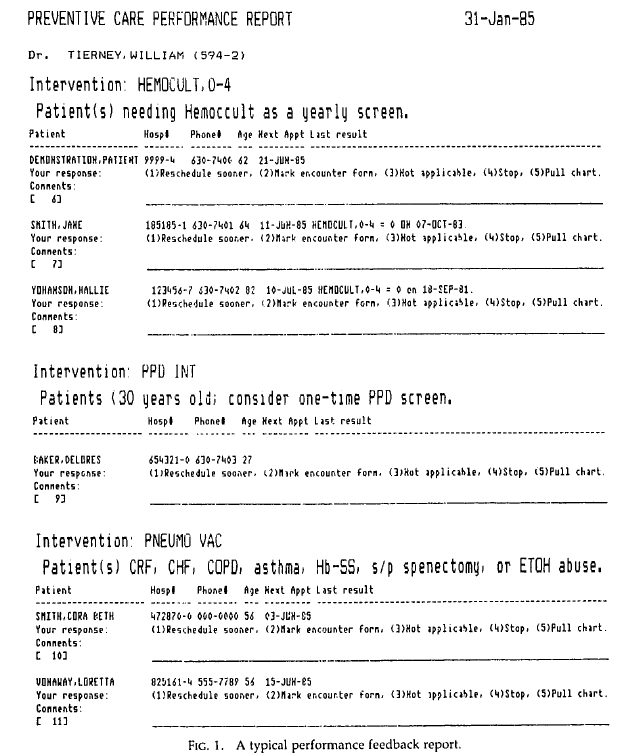


**Things to think about as you reflect on the A&F intervention**

- Given your theoretical expertise, how do you think the intervention would work and/or how would you go about improving an intervention like this?

Let’s focus on a particular hypothesis your theory suggests…

- What would that be? Can you frame a clear **hypothesis** for us?
- What is the **theoretical mechanism of action**? (i.e. what makes this effective?)
- On what **outcomes** would this hypothesis show its effects?
- Are there any **contextual factors** that would affect the hypothesis? (i.e. for whom and under what circumstances would this be effective?)
- Can you identify a specific **theory or model** that motivates this hypothesis?
- Are there any **specific readings** you would point us to about these ideas?

*What else?*

- **Reminder**: The goal of this exercise is *to* generate ***theory- informed hypotheses*** (as opposed to just general ideas) that you think should be considered in improving this kind of intervention.

Are there any **other ideas/concepts** that you think might be relevant to these kinds of interventions?

- Can you identify a specific **theory** that motivates this hypothesis?
- Are there any **specific readings** you would point us to about these ideas?
- On what **outcomes** would this hypothesis show its effect?
- Are there any **contextual factors** that would affect the hypothesis? (i.e. for whom and under what circumstances would this be effective?)
- What is the **theoretical mechanism of action** by which this should work?

Are there **any people/theory experts** that you can think of that might have important input into these issues?

Thank you very much for this.

As mentioned in our original email, we are going to be going through a process of trying to operationalize ideas from these sessions into hypotheses about feedback interventions that are as concrete and testable as possible. In a few days, we’d like to send you another email with a document that summarizes the ideas you’ve given us. We’ll try to be as clear as we can, but we would like you to have a look at what we do, see if you agree with how we have operationalized your ideas, and see if you can add any more detail/clarity to our work.

We’ll try to keep it short; it should take from 30-60 minutes at most. Is it ok for us to send you this document and get your feedback on it?

**Thanks for your help!**
