# Supplementary material for: Advancing the literature on designing audit and feedback interventions: identifying theory-informed hypotheses
Source: Implement Sci. 2017 Sep 29;12:117. doi: 10.1186/s13012-017-0646-0 (PMC5622490; doi:10.1186/s13012-017-0646-0)
Supplement: Supplementary file 2 — All 313 hypotheses organized in 30 themes. (DOCX 37 kb) [file 13012_2017_646_MOESM2_ESM.docx]

Additional file 2: Hypotheses organized in 30 themes.

| **Themes**  **(N = 30)** | **# of Hypotheses** (N = 313) | **Example Hypotheses**  A&F/A&F interventions will be more effective… |
| --- | --- | --- |
| **Related to the Recipient** |  |  |
| 1. Trust/Credibility | 14 | if it is perceived to be without conflict of interest; when recommendations related to the A&F are based on good quality evidence |
| 1. Motivation/Intention | 13 | if it is accompanied with positive reinforcement to those who have improved their performance; when accompanied by incentive |
| 1. Recipient Characteristics | 9 | for those with a mastery goal orientation if it involves comparison to others |
| 1. Recipient Priorities | 9 | when targeted at behaviours that the target feels is important to their professional roles/responsibilities |
| 1. Attack on Self-Identity | 7 | when measures are used to prevent a defensive response (providing other ‘reassuring’ messages as well) |
| 1. Attract/Maintain Attention | 6 | if they engage the target’s attention |
| 1. Self-Efficacy/Control | 5 | if the behaviour is under the control of the recipient |
| **Related to the Behaviour** |  |  |
| 1. Remove Barriers | 11 | if they address barriers to change in behaviour |
| 1. About Aspects of Behaviour | 7 | for behaviors that are easy compared to those that are harder to do |
| 1. Decision Processes or Conceptual Model | 4 | if designed with a clear understanding of the decision making process underlying the behaviour to be changed |
| **Related to the Content of the A&F** |  |  |
| 1. Cognitive Load | 33 | if as few graphs as possible are presented; without unnecessary depth elements; if the graphical representations are clearly and consistently labelled; when colour changes are purposeful and convey meaning; when presenting absolute numbers as opposed to percentages; when graphical clutter is removed; when focused on a few, most important behaviours; |
| 1. Comparisons | 26 | when the benchmark comparison is justified to be a reasonable standard; when a comparator is provided; when multiple individual practice data is presented along with the recipient’s data; if it involves a comparison to the self; if the comparator is specific to the recipient’s own context/practice. |
| 1. Action Plans/Coping Strategies | 19 | if clear direction on how to change behaviour is provided |
| 1. A&F Specificity | 16 | if individual level provider data is provided; if patient-specific information is provided; if it is as specific as possible |
| 1. Goal Setting | 16 | if it is accompanied by a goal that is specific |
| 1. Justify Need for Behaviour Change | 10 | if accompanied by evidence supporting the behaviour change |
| 1. Cognitive Influences | 7 | if emphasis is on what needs to be achieved (loss framing) as opposed to what was achieved (gain framing). |
| 1. Nature of the Data | 6 | if graphical representation displays the variability of data in order to indicate the error or uncertainty |
| 1. Guide Reflection | 6 | if it involves a personal reflection component |
| 1. Improving Memory | 6 | if the reminder messages are presented in real time/point of care; if incorporates an emotional message underlining the desired behaviour. |
| **Related to the Delivery of the A&F** |  |  |
| 1. A&F Timing | 20 | if individual change data over time is provided; when presented multiple times; when presented at the time of decision making |
| 1. Social Engagement | 17 | if they involve engaging recipients in social discussion about the A&F |
| 1. Knowledge/Learning | 13 | if it creates opportunities to learn |
| 1. User-Guided Experience | 6 | when complex information is scaffolded to allow a recipient to get more information if and when they want |
| 1. In-Person A&F | 2 | when provided with human contact |
| 1. Responding to A&F Providers | 2 | if they allow the recipient an opportunity to indicate why a recommended action was not taken. |
| **OTHER** |  |  |
| 1. Opportunity Costs | 7 | when there are few costs to change behaviour |
| 1. Environment | 4 | if the environment encourages the desired behaviour as the default. |
| 1. Development Process Involvement | 2 | when recipients have been involved in the design of the A&F |
| 1. Single Hypotheses | 10 | If they imply some kind of extended commitment; if the recipient generates a response immediately prior to receiving the A&F; if the goal is made public; if it is provided to the intended target for behavior change; it includes multiple modes of information (e.g. pictures and text) |
